# Salidroside suppresses gastric cancer progression via miR-1343-3p-mediated repression of ACOT11 and disruption of fatty acid metabolism

**DOI:** 10.3389/fonc.2025.1651857

**Published:** 2025-09-04

**Authors:** Zhendong Zhang, Mingyuan Cao, Yuxin Du, Pingyi Wang, Xinrui Hou, Xiaoping Wang

**Affiliations:** ^1^ School of Medicine, Xizang Minzu University, Xianyang, Shaanxi, China; ^2^ Key Laboratory of High Altitude Hypoxia Environment and Life Health, Xizang Minzu University, Xianyang, Shaanxi, China; ^3^ Department of Rehabilitation Medicine, The Third Affiliated Hospital, Sun Yat-sen University, Guangzhou, Guangdong, China

**Keywords:** gastric cancer, salidroside, lipid metabolism, miR-1343-3p, ACOT11

## Abstract

**Objective:**

Salidroside, a bioactive compound derived from Rhodiola, has been demonstrated to upregulate the tumor suppressor miR-1343-3p, leading to suppression of gastric cancer growth. However, the precise molecular mechanisms underlying salidroside-mediated regulation of lipid metabolism via miR-1343-3p and its downstream mRNA targets remain poorly understood.

**Methods:**

The interaction between miR-1343-3p and ACOT11 was evaluated through Pearson correlation analysis, sequence-based binding site alignment, and RNA immunoprecipitation (RIP) assays. The effects of salidroside treatment on cell proliferation, gene and protein expression, downstream metabolites, and energy production were assessed through a series of *in vitro* and *in vivo* experiments, including the CCK-8 assay, colony formation assay, RT-qPCR, Western blot, ELISA, cell transfection, and xenograft tumor models.

**Results:**

The expression of miR-1343-3p is negatively correlated with ACOT11 mRNA, which is closely associated with lipid metabolism. Salidroside significantly inhibits the proliferation of gastric cancer cells in a dose-dependent manner. Compared to untreated controls, salidroside-treated gastric cancer cells showed decreased ACOT11 mRNA/protein expression but increased miR-1343-3p levels. This was accompanied by elevated substrate fatty acyl-CoA concentrations with concurrent reductions in acetyl-CoA, FFA, and ATP. ACOT11 is a downstream target of miR-1343-3p, up-regulating miR-1343-3p expression reduces ACOT11 expression, while down-regulating miR-1343-3p expression increases ACOT11 expression. *In vivo*, salidroside significantly inhibited tumor growth in gastric cancer xenograft models.

**Conclusions:**

We demonstrate that salidroside exerts anti-proliferative effects in gastric cancer by targeting the miR-1343-3p/ACOT11/FFA lipid metabolism signaling pathway, disrupting cancer cell energy production. These regulatory factors hold promise as novel therapeutic targets for gastric cancer.

## Graphical Abstract

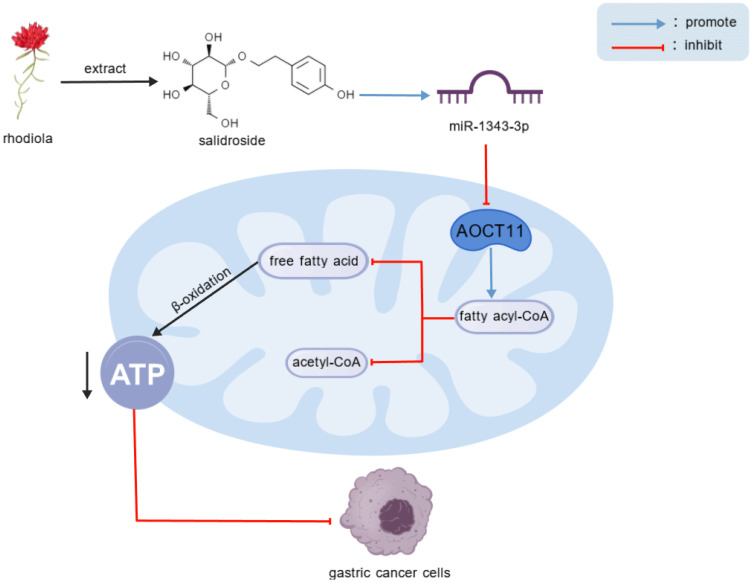

Salidroside, an extract of Rhodiola, upregulate miR-1343-3p transcription, leading to the targeted down-regulation of ACOT11 expression. This results in the accumulation of fatty acyl-CoA, reduced hydrolysis, decreased production of FFA and acetyl-CoA, interference with lipid metabolism and energy production in gastric cancer cells, and ultimately inhibits the proliferation of gastric cancer cells.

## 1 Introduction

Gastric cancer (GC) represents a leading cause of global cancer-related morbidity and mortality, accounting for substantial disease burden worldwide. Current surveillance statistics classify GC as China’s fifth most common cancer diagnosis and third leading contributor to cancer mortality, underscoring its disease burden (1). The limited durability of therapeutic response and poor target specificity of conventional chemotherapeutics contribute substantially to tumor recurrence and metastatic progression, ultimately driving unfavorable patient survival outcome. Therefore, there is an urgent need to identify potential drug targets to improve the prognosis of GC patients.

Salidroside, the main active component of the Tibetan medicine Rhodiola (2), has been shown to possess various effects, including immune regulation, hypoxia inhibition, metabolic suppression, anti-aging, anti-diabetic, and anti-tumor properties (3–5). As a multi-effect and multi-target biological regulator, salidroside has been confirmed to exert anti-tumor effects by inducing cancer cell apoptosis and cell death. For example, salidroside can promote apoptosis of human gastric cancer AGS cells by inhibiting the PI3K/AKT signaling pathway (6), inhibit gastric cancer progression by inhibiting sugar fermentation, or inhibit gastric cancer proliferation (7) and inhibit the occurrence and development of gastric cancer cells by inducing ferroptosis (8). However, the other potential anti-tumor mechanisms of salidroside in GC, such as lipid metabolism, remain to be fully elucidated.

Recent studies have revealed that reprogramming lipid metabolism plays a crucial role in the proliferation and migration of cancer cells, and its lipid metabolites can also alter the tumor microenvironment (9). Research has shown that abnormal lipid metabolism plays an important role in the development and progression of GC, participating in cancer cell membrane biosynthesis, forming signaling pathways, promoting protein-protein interactions, and facilitating angiogenesis and tumor metastasis (10). As a critical energy source for cells, lipids fuel tumor cell metabolism, reshape the tumor microenvironment, and suppress immune cell activity. Additionally, they contribute to drug resistance in cancer cells, thereby driving GC progression, invasion, metastasis, and rapid proliferation (11, 12). Especially fatty acids can provide energy for tumor cells and participate in the biosynthesis of cell membrane lipids and signal transduction molecules (13). Mesenchymal stem cell-induced lncRNA Hcp 5 drives fatty acid oxidation through miR-3619-5p/AMPK/PGC1α/CEBPB axis, promoting chemotherapy resistance in GC cells, indicating that targeting Hcp 5 is a new approach to enhance the efficacy of GC chemotherapy drugs (14). Thus, elucidating the molecular mechanisms underlying dysregulated lipid metabolism in GC progression is crucial for understanding its proliferative and metastatic behavior—and for advancing targeted therapeutic strategies.

Advances in molecular biology have revealed that miRNA-mediated lipid metabolism reprogramming in GC cells regulates key signaling pathways, driving tumor proliferation and migration (15). Recent research has discovered that fatty acid amide hydrolase promotes GC progression by regulating arachidonoylethanolamide/lysophosphatidic acid signaling and activating the cyclooxygenase-2/prostaglandin E2 axis. Further studies have revealed that miR-1275 may indirectly regulate these lipid signaling pathways by targeting fatty acid amide hydrolase, thereby influencing GC progression (16). Research indicates that the Human histocompatibility leukocyte antigen complex p5 acts as a molecular sponge for miR-3619-5p, leading to the upregulation of PPARG coactivator 1 alpha. This promotes the formation of the PGC1α/CEBPB transcriptional complex, which transcriptionally induces carnitine palmitoyltransferase 1, thereby enhancing fatty acid oxidation and driving gastric cancer progression (14). These findings suggest that lipid metabolism reprogramming is intricately and closely linked to the proliferation, invasion, and metastasis of GC, involving multiple signaling pathways regulated by key enzymes associated with abnormal lipid metabolism. Fundamentally, in cancer cells, lipid metabolism reprogramming appears to be driven by miRNA-mediated dysregulation of key metabolic enzymes (17). Therefore, identifying critical miRNA-regulated enzymes in lipid metabolic reprogramming is essential for elucidating GC progression and developing targeted therapies.

The tumor suppressor miR-1343-3p is a short non-coding RNA that mediates post-transcriptional gene regulation through targeted mRNA binding, thereby controlling mRNA stability and translation (18). Our previous research has shown for the first time that salidroside exerts potent anti-tumor effects by selectively upregulating miR-1343-3p, such as leading to direct suppression of oncogenic MAP3K6 and MMP24 signaling pathways and consequent inhibition of GC progression (18). There are also studies showing that as an anti-cancer factor, miR-1343-3p can inhibit the expression and activation of the GC oncogene TEA domain transcription factor 4 (TEAD4) (19). However, the key nodes of lipid metabolism and gene regulatory mechanisms by which salidroside inhibits GC growth remain unclear. Therefore, this study investigates miR-1343-3p as a central regulator to identify salidroside’s key lipid metabolism targets in GC. By elucidating the miR-1343-3p/ACOT11/FFA signaling axis, we aim to uncover novel mechanistic insights and advance targeted therapeutic strategies for GC treatment.

## 2 Methods

### 2.1 Cell culture and solution preparation

The human GC cell line MGC-803 was purchased from Nanjing Biory Biotechnology Co., Ltd. (Nanjing, China). The MGC-803 cells were cultured in RPMI-1640 medium (Gibco, USA) supplemented with 10% fetal bovine serum (Evergreen, China) and 1% penicillin-streptomycin (Hyclone, USA). The human GC cell line AGS was purchased from Suzhou Haixing Biotechnology Co., Ltd. (Suzhou, China). The AGS cells were cultured in Ham’s F12K medium (Haixing, China) supplemented with 10% fetal bovine serum (Haixing, China) and 1% penicillin-streptomycin (Hyclone, USA). All cells were maintained in a humidified incubator (Thermo, USA) at 37 °C with 5% CO_2_. When the cells reached approximately 80-90% confluency, they were detached using 0.25% trypsin (Vivocell, China) for subculturing or plating. All experimental procedures were performed within 8 passages of the cells.

Salidroside (Solarbio, China) was dissolved in RPMI-1640 medium at concentrations of 2, 4, 6, 8, and 10 μmol/mL for MGC-803 cells subsequent experiments and dissolved in Ham’s F12K medium at concentrations of 0.01, 0.02, 0.04 and 0.08 μmol/mL for AGS cells subsequent experiments. The group without salidroside treatment served as the control group.

### 2.2 CCK-8 and colony formation assays

In a 96-well plate, 100 μL of GC cells at a density of 0.25×10^4^ were evenly seeded per well. After the cells adhered, salidroside was added at concentrations of section 2.1 , respectively. The cells were then cultured for an additional 24 and 48 hours, after which CCK-8 (Boster, China) reagent was added to assess the viability of GC cells treated with different drug concentrations. The absorbance at 450 nm was measured using a microplate reader (Thermo, USA), and the results were calculated and plotted. Cell viability was assessed using the following formula. Cell viability (%) = (absorbance of the experimental group/absorbance of the control group) ×100%.

In a 12-well plate, 100 μL of GC cells at a density of 300 were evenly seeded per well. After the cells adhered, salidroside was added at concentrations of 4, 6, and 8 μmol/mL for MGC-803 cells. After 48 hours, the medium was replaced, and the cells were cultured for an additional 1–3 weeks or until the majority of individual clones contained more than 50 cells. The cells were then fixed with 4% paraformaldehyde at room temperature, stained with 0.1% crystal violet staining solution, washed several times with PBS, air-dried, photographed, and the colonies were counted.

### 2.3 Prediction of miR-1343-3p-lipid metabolism target mRNAs

In the MGC-803 cells sequencing RNA database previously established by the research group, miR-1343-3p-mRNA target gene comparisons were integrated with GO and KEGG enrichment analyses. The associations between them were assessed using Pearson correlation and gene locus analysis (details on the database, https://doi.org/10.1080/15384047.2024.2322206, establishment and methodology can be found in prior research (18)). GEPIA database (http://gepia.cancer-pku.cn/) analysis of acyl-CoA thioesterase 11 (ACOT11) expression in GC tissue.

### 2.4 RNA binding protein immunoprecipitation assay

The target protein antibody IP group and the negative control IgG group were set up. An appropriate amount of Protein A/G Agarose (40 μL, Beyotime, China) was added, washed, and resuspended using NT2 Wash Buffer (Beyotime, China). Then, ACOT11 (2 μg, a mouse antibody of Santa, USA, cat. No. sc-398738) and Mouse IgG (2 μg, Beyotime, China, cat. No. A7028) were added separately, followed by incubation at room temperature for 30 minutes. After centrifugation at 4 °C, 1000 g for 1 minute, the supernatant was discarded to obtain pre-bound Protein A/G Agarose. Cells were lysed using Lysis Buffer (Beyotime, China), and a portion of the supernatant (10% cracking solution from a RIP reaction) was taken as Input for subsequent detection. The remaining supernatant was added to the pre-bound Protein A/G Agarose and incubated on a shaker at 4 °C for 4 hours. An appropriate amount of Elution Buffer (Beyotime, China) was then added and mixed, followed by incubation at 55 °C for 30 minutes. After incubation, total RNA was extracted using Trizol (AG, China). The extracted RNA was reverse-transcribed into cDNA using a reverse transcription kit (Sparkjade, China). Finally, the miR-1343-3p gene fragment was amplified using SYBR Green qPCR Mix (Sparkjade, China), and gene expression levels were detected. The reverse transcription conditions for miR-1343-3p were as follows: 25 °C for 5 minutes, 50 °C for 15 minutes, and 85 °C for 5 minutes. The qPCR conditions were: 94 °C for 3 minutes, 94 °C for 10 seconds and 60 °C for 30 seconds, followed by 40 cycles. The melting curve was collected using the instrument’s default program (Bio-Rad, USA). The 2^-△△^Ct method was used to calculate the enrichment fold of miR-1343-3p in RIP relative to the negative control. Primer sequences are listed in **Table 1**.

**Table 1:** qPCR primer sequences.

Gene	Primer sequences (5’→3’)
miR-1343-3p	F: CGCGGCCTTAATGCTAATTGTGA R: AGTGCAGGGTCCGAGGTATT
ACOT11	F: TGACAAGTTCCTCTCCTTCCACATG R: TGGCGTCGTCCTCGTCTACC
U6	F: GCTTCGGCAGCACATATACTAAAAT R: CGCTTCACGAATTTGCGTGTCAT
GAPDH	F: TGACATCAAGAAGGTGGTGAAGCAG R: GTGTCGCTGTTGAAGTCAGAGGAG

### 2.5 Real-time RT-PCR

The method for total RNA collection and the detection of miR-1343-3p and ACOT11 expression levels were performed as described from 2.4. U6 and GAPDH were used as internal references for miR-1343-3p and ACOT11, respectively. The reverse transcription conditions for ACOT11 were as follows: 50°C for 15 minutes and 85°C for 5 seconds. The 2^-△△^Ct method was used to calculate the relative expression levels of each gene. Primer sequences are listed in **Table 1**.

### 2.6 Western blot

Collect the supernatant of the culture medium (store in a -80°C freezer for future use). After fully lysing the cells using RIPA Lysis Buffer (Beyotime, China), after centrifugation at 4 °C, 12000 g for 5 minutes, the protein supernatant was collected, and the protein concentration was measured using a BCA protein assay kit (Boster, China). Protein samples were subjected to SDS-polyacrylamide gel electrophoresis and transferred onto a PVDF membrane (Millipore, USA). The membrane was blocked with 5% BSA solution (Solarbio, China) for 2 hours. Primary antibodies, including ACOT11 (1: 2000, a rabbit antibody of Proteintech, China, cat. No. 10776-1-AP) and GAPDH (1: 1250, Boster, China, cat. No. BM3874), were incubated with the membrane at 4°C overnight. Subsequently, secondary antibodies, Rabbit (HRP) (1: 10000, abcam, USA, cat. No. ab6721), were incubated with the membrane at room temperature for 2 hours. The ultra-sensitive ECL chemiluminescence reagents A and B (Kermey, China) were mixed in a 1: 1 ratio and applied to the membrane. The membrane was then visualized and imaged using a chemiluminescence gel imaging system (Bio-Rad, USA).

### 2.7 Detection of fatty acyl-CoA, acetyl-CoA and free fatty acid concentration

Following the instructions of the enzyme-linked immunosorbent assay (ELISA) kit (MEIMIAN, China), the absorbance of fatty acyl-CoA (FA-CoA) and acetyl-CoA (A-CoA) in the cell supernatant under different treatments (Collected in Method 2.6) was measured at 450 nm using a microplate reader, and the sample concentration was calculated according to the standard curve formula, all tests were repeated three times. In accordance with the free fatty acid assay kit (Nanjingjiancheng, China) instructions, the absorbance of free fatty acids (FFA) in the cell supernatant under different treatments was measured at 546 nm using a microplate reader. FFA was evaluated using the following formula. $\text{FFA }\left(\text{μmol}/\text{L}\right)=\frac{△\text{A samples}-△\text{A blanks}}{△\text{A standards}-△\text{A blanks}}$ ×C_calibration_×1000. C_Calibration_: Standard concentration, 1.04 mmol/L.

### 2.8 Detection of ATP concentration

Collect GC cells at 80-90% confluence, seed them evenly in six-well plates, and after cell attachment, add drug-free medium (control group) and low, medium, and high-dose salidroside (4, 6, and 8 μmol/mL salidroside solutions for MGC-803 cells, 0.02, 0.04, and 0.08 μmol/mL salidroside solutions for AGS cells ). After fully lysing the cells using the ATP assay lysis buffer from the ATP detection kit (Beyotime, China), the supernatant was collected by centrifugation at 4 °C, 14000 g for 10 minutes. In an opaque black 96-well plate, 100 μL of ATP detection working solution was added to each well and allowed to stand at room temperature for 3–5 minutes. Then, 20 μL of the sample or standard was added to each well, mixed thoroughly, and the relative luminescence units were measured using a multifunctional cell imaging detection system (Biotek, USA). The sample concentration was calculated based on the standard curve formula.

### 2.9 Cell transfection

miR-1343-3p mimic, NC mimic, miR-1343-3p inhibitor, NC inhibitor, si-ACOT11 and si-NC (Biomics, China) were transfected into MGC-803 cells using Lipofectamine™ 2000 (Invitrogen, USA). Primer sequences are listed in **Table 2**. The final concentrations for transfection were 50 nM for miR-1343-3p mimic and NC mimic, and 100 nM for miR-1343-3p inhibitor, NC inhibitor, si-ACOT11 and si-NC. When combined with salidroside, the concentration of salidroside used was the IC_50_ value.

**Table 2:** Cell transfection primer sequences.

Gene	Primer sequences (5’→3’)
miR-1343-3p mimic/miR-1343-3p agomir	sense: CUCCUGGGGCCCGCACUCUCGCUU antisense: AAGCGAGAGUGCGGGCCCAGGAG
NC mimic	sense: UCACAACCUCCUAGAAAGAGUAGA antisense: UCUACUCUUUCUAGGAGGUUGUGA
miR-1343-3p inhibitor	antisense: AAGCGAGAGUGCGGGCCCCAGGAG
NC inhibitor	antisense: UCUACUCUUUCUAGGAGGUUGUGA
si-ACOT11	sense: GAGAGAUCACCAAGGUGAAdTdT antisense: UUCACCUUGGUGAUCUCUCdTdT
si-NC	sense: UUCUCCGAACGUGUCACGUdTdT antisense: ACGUGACACGUUCGGAGAAdTdT

### 2.10 Xenograft tumor model

This study was reviewed and approved by the Ethics Committee of Xizang Minzu University, with approval number 2024-014. The animal experiments complied with the ARRIVE guidelines. The animals used in this study were SPF-grade nude mice, female, 4 weeks old, with a body weight of approximately 15–20 grams, purchased from Jiangsu Huachuang Xinnuo Pharmaceutical Technology Co., Ltd. (Jiangsu, China), with a production license number of SCXK (Su) 2020–0009 and an animal qualification certificate number of 320928240100203557. The entire breeding process strictly adheres to SPF-grade animal facility operating standards, with free movement, feeding, and drinking. Environmental parameters are maintained at a temperature of 22 ± 1°C, humidity of 55 ± 5%, and a 12-hour light/dark circadian rhythm.

After one week of acclimatisation under identical conditions and locations, the nude mice were randomly divided into three groups: the model control group, the salidroside group, and the miR-1343-3p overexpression group (primer sequences are listed in **Table 2**), with 5 mice in each group and a total of 15 mice. Following the ear tag order, the left axillary skin of the nude mice was disinfected with 75% ethanol, and a suspension of MGC-803 cells (2×10^6^ cells/mouse) was subcutaneously injected into the left axillary. Observe tumor formation in nude mice, measure tumor volume using vernier calipers, and initiate drug administration when the tumor volume reaches approximately 100 mm^3^.The model control group and the salidroside group were intraperitoneally injected with saline (0.2 mL/20g) and salidroside (100 mg/kg, dissolved in saline), respectively, administered every 2 days. The miR-1343-3p over-expression group received local administration of miR-1343-3p agomir (3 mg/kg, Biomics, China, primer sequences are listed in **Table 2**) at the tumor site, twice a week. Tumor volume (V=0.5×L×W^2^) was calculated weekly for 4 consecutive weeks. After 4 weeks, the nude mice were euthanized painlessly, and the tumor tissues were weighed.

### 2.11 Statistical methods

The experimental data of IC_50_ were analyzed and plotted using GraphPad Prism 9.5 software (https://www.graphpad.com/) and other experimental datas were analyzed and plotted using R version 4.2.1 (https://www.r-project.org/). The results are expressed as mean ± standard deviation. Comparisons between two groups were performed using the *t*-test. For comparisons among multiple groups, if the data met the assumptions of normality and homogeneity of variance, One-way ANOVA was used; if the data met normality but not homogeneity of variance, Welch’s one-way ANOVA was applied. A *P*< 0.05 was considered statistically significant (*P< 0.05, **P< 0.01, ***P< 0.001). All the tests were performed three times.

## 3 Results

### 3.1 Salidroside inhibited the viability and proliferation of GC cells

**Figure 1:**
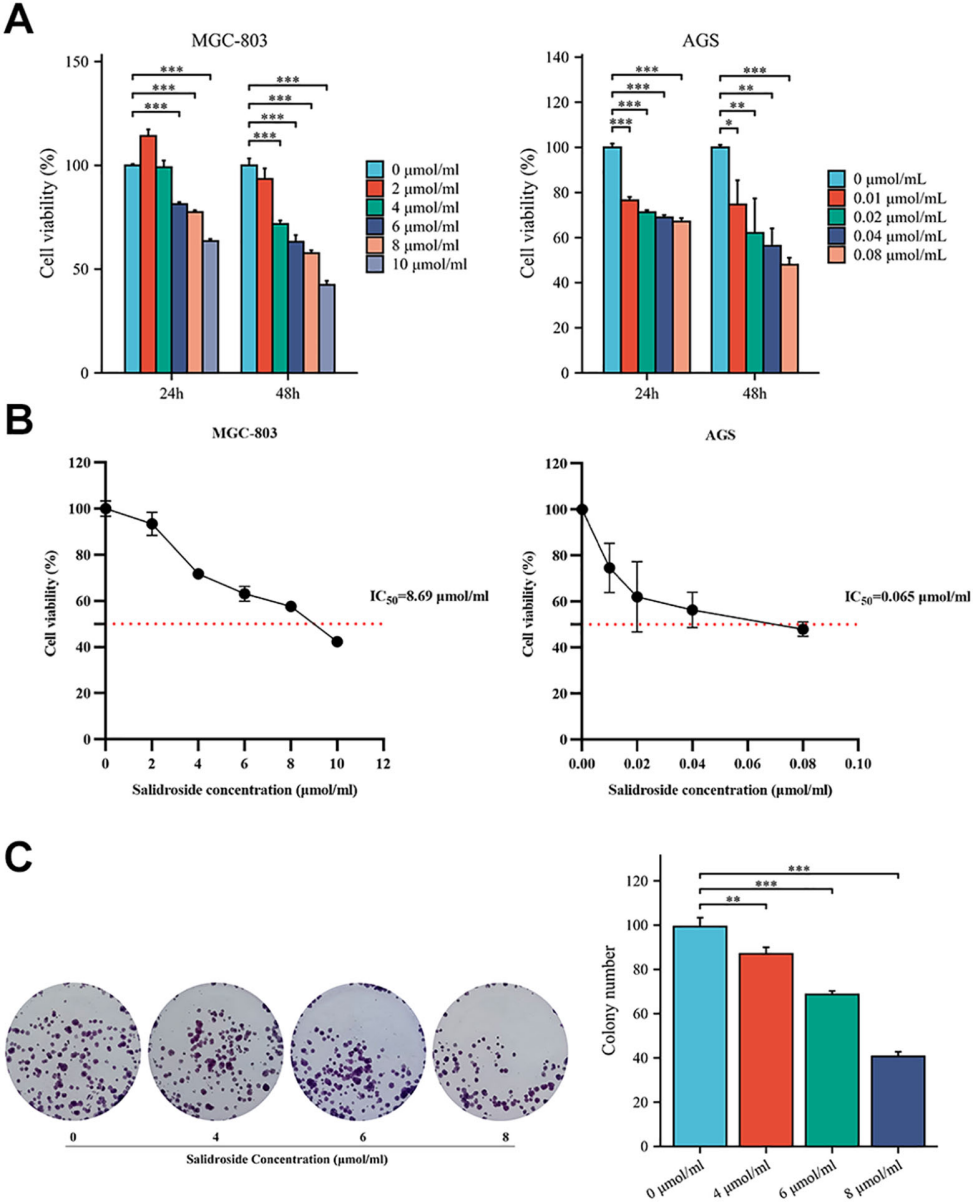
Salidroside inhibits the proliferation of GC cells. **(A)**. The CCK-8 assay demonstrated that salidroside inhibits the proliferation of GC cells in a time-dose dependent manner (*n*=3); **(B)**. Cell Vitality Line Chart of 48 hours (*n*=3). **(C)**. The colony formation rate was decreased after salidroside treatment in MGC-803 cells for 48 hours, indicating inhibited proliferation capacity (*n*=3); *P<0.05, **P< 0.01, ***P< 0.001. The group without salidroside treatment served as the control group (0 μmol/mL).

To determine the inhibitory effects of salidroside on GC cell viability and proliferation, we performed CCK-8 and colony formation assays. CCK-8 viability assays revealed that salidroside exerted anti-proliferative effects in GC cells. In MGC-803 cells, treatment with 6-10 μmol/mL salidroside for 24 hours significantly reduced cell viability, while extended 48-hour exposure demonstrated enhanced efficacy, showing significant growth inhibition across all tested concentrations (2-10 μmol/mL). In AGS cells, treatment with 0.01-0.08 μmol/mL salidroside for 24 hours significantly reduced cell viability, while extended 48-hour exposure demonstrated enhanced efficacy. The results demonstrate that the inhibitory effect of salidroside was more potential at 48 hours compared to 24 hours (**Figure 1A**). Therefore, based on the IC_50_ concentrations of salidroside treatment in MGC-803 cells (8.69 μmol/mL) and AGS cells (0.065 μmol/mL) at 48 hours (**Figure 1B**), we selected salidroside concentrations of 4, 6, and 8 μmol/mL (low, medium, and high-dose salidroside groups) for treating MGC-803 cells and 0.02, 0.04, and 0.08 μmol/mL (low, medium, and high-dose salidroside groups) for treating AGS cells for 48 hours as the conditions for subsequent experiments. The colony formation assay results demonstrated that the colony formation rate significantly decreased in the treated group, demonstrating potent anti-proliferative effects (**Figure 1C**). These results demonstrate that salidroside suppresses GC cell viability and proliferation in time- and dose-dependent manners.

### 3.2 Screening of target genes: identifying key lipid metabolism effectors influencing GC cell proliferation

**Figure 2:**
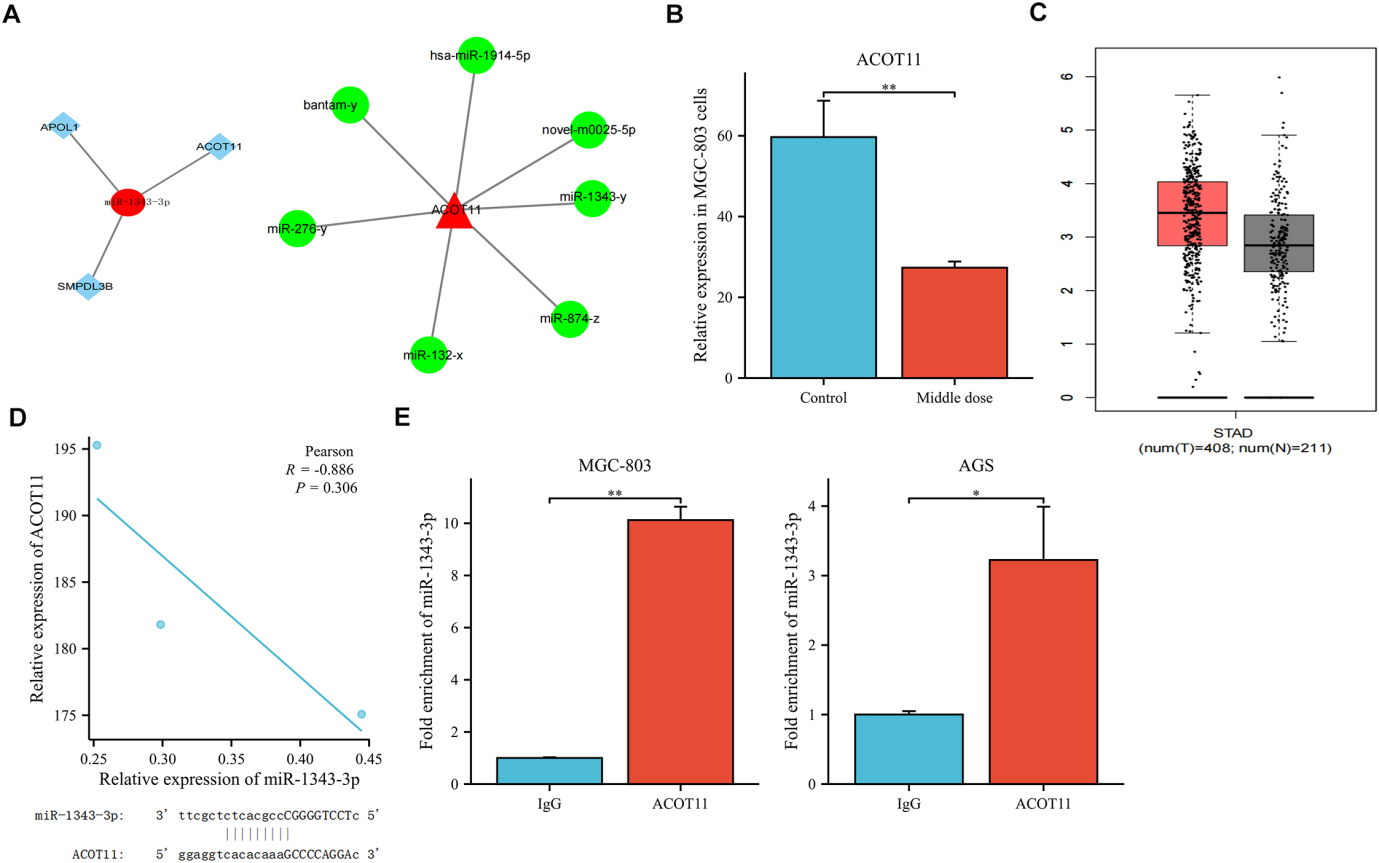
Co-relation between miR-1343-3p and ACOT11. **(A)**. Interaction plots of ACOT11 with miR-1343-3p are shown. **(B)**. High-throughput sequencing was used to detect alterations in ACOT11 expression in human MGC-803 cells following treatment with salidroside(*n*=3); **(C)**. Differential expression analysis of ACOT11 in GC tissues in GEPIA database; **(D)**. Pearson correlation and gene locus analysis were performed to assess the association between ACOT11 and miR-1343-3p in human MGC-803 cells (*n*=3); **(E)**. RNA binding protein immunoprecipitation assay was conducted to validate the interaction between ACOT11 and miR-1343-3p (*n*=3); *p< 0.05, **P< 0.01.

Previous research results indicated that a total of 1873 miRNAs were expressed based on high-throughput sequencing results. Compared with the control group, there were significant differences in the expression levels of 44 miRNAs after treatment with salidroside (absolute difference fold ≥ 2, P<0.05). Among them, the tumor suppressor factor miR-1343-3p is nearly non-expressed in the control group of MGC-803 cells but is significantly upregulated after treatment with salidroside (18). Through integrated bioinformatic analysis combining miRNA-mRNA target prediction, Gene Ontology (GO) annotation, and KEGG pathway enrichment (18), three lipid metabolism-related target mRNAs were identified to be negatively regulated by miR-1343-3p (**Figure 2A**). Among these, ACOT11—a key enzyme for FFA production—showed the highest expression in MGC-803 cells and was downregulated by over 2-fold after salidroside treatment (**Figure 2B**). Further analysis using the GEPIA database (http://gepia.cancer-pku.cn/) revealed that, compared to normal tissues, ACOT11 is highly expressed in GC tissues (**Figure 2C**). Therefore, we identified ACOT11 as a key target closely associated with miR-1343-3p regulation and significantly downregulated, showing a negative correlation with miR-1343-3p expression. Further Pearson correlation and gene locus analyses demonstrated that ACOT11 and miR-1343-3p have specific binding sites and a clear association in MGC-803 cells (**Figure 2D**). RIP experiments revealed that the relative expression level of miR-1343-3p in the IP group was significantly higher than that in the IgG group, indicating that miR-1343-3p was significantly enriched in the ACOT11 immunoprecipitation samples. This suggests that ACOT11 may be regulated by miR-1343-3p, thereby affecting lipid metabolism and tumor progression (**Figure 2E**). These findings suggest that salidroside likely inhibits GC cell proliferation by down-regulating ACOT11 through miR-1343-3p targeting, thereby interfering with lipid metabolism and energy production in GC cells.

### 3.3 Salidroside upregulates miR-1343-3p expression, down-regulates ACOT11 expression, and inhibits lipid metabolism and energy production in GC cells

**Figure 3:**
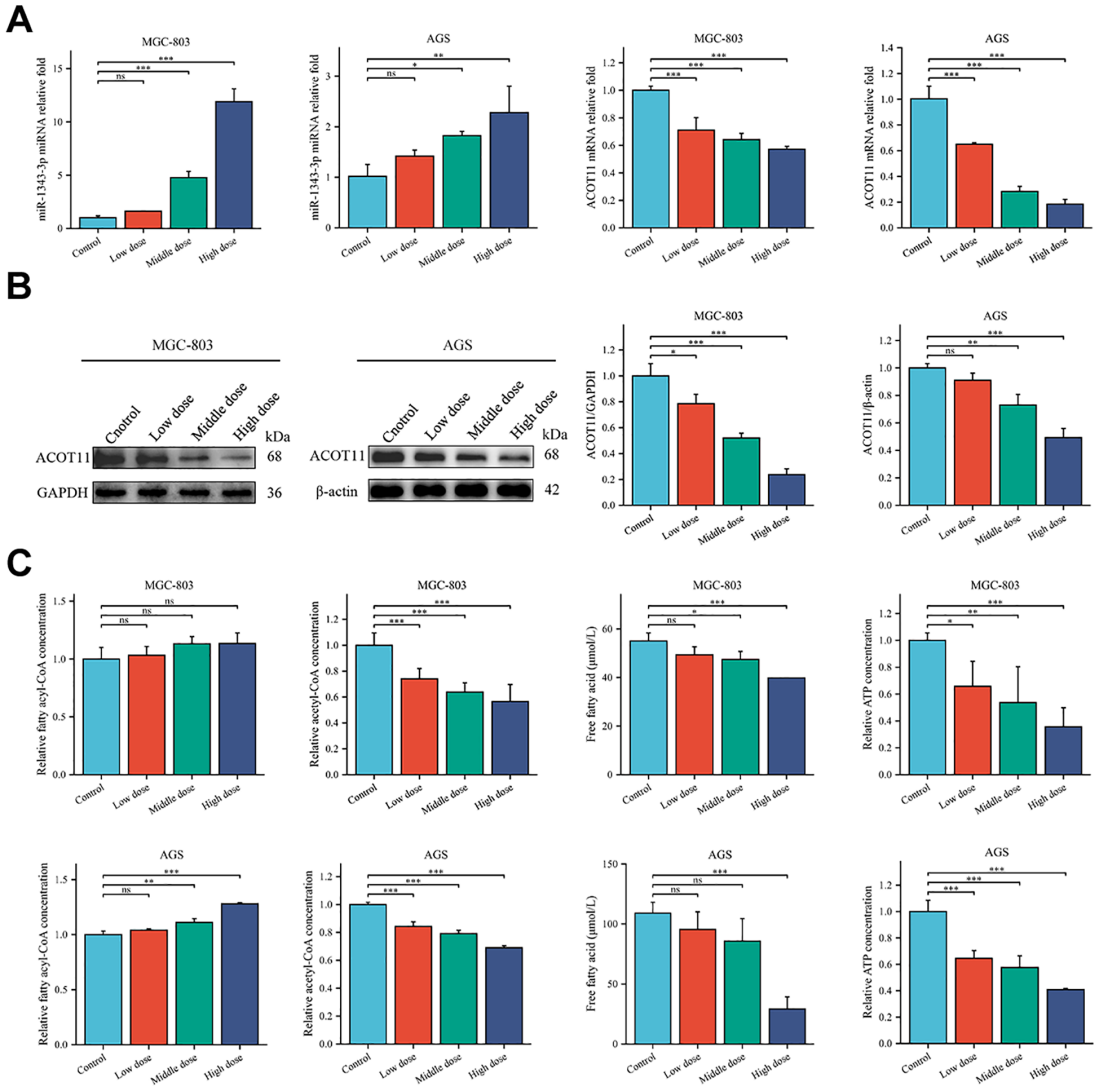
Salidroside suppresses GC progression by disrupting lipid metabolic pathways and inhibiting cellular proliferation. **(A)**. RT-qPCR analysis of the gene expression levels of miR-1343-3p and ACOT11 in GC cells (*n*=3); **(B)**. Western blot analysis of the protein expression levels of ACOT11 in GC cells (*n*=3); **(C)** ELISA, microplate assay, and luciferase assay were used to measure the concentration changes of fatty acyl-CoA, acetyl-CoA, FFA, and ATP in GC cells (*n*=3); *P< 0.05, **P< 0.01, ***P< 0.001. MGC-803: Control is 0 μmol/mL; Low dose is 4 μmol/mL; Middle dose is 6 μmol/mL; High dose is 8 μmol/mL; AGS: Control is 0 μmol/mL; Low dose is 0.02 μmol/mL; Middle dose is 0.04 μmol/mL; High dose is 0.08 μmol/mL.

RT-qPCR was used to validate the changes in gene expression of miR-1343-3p, ACOT11, and other target molecules in GC cells following salidroside intervention. The results showed that, compared to the control group, the expression level of miR-1343-3p was upregulated, while the mRNA expression level of ACOT11 was downregulated in GC cells treated with salidroside (**Figure 3A**). Western blot analysis was performed to assess salidroside-induced alterations in ACOT11 expression and its downstream signaling effectors in GC cells. The results indicated that, compared to the control group, the protein expression level of ACOT11 was reduced in GC cells treated with salidroside (**Figure 3B**). To verify the effects of salidroside treatment on lipid synthesis and energy levels in GC cells, ELISA, microplate assay, and luciferase assay were used to measure the relative concentrations of fatty acyl-CoA, acetyl-CoA, FFA, and ATP in GC cells after salidroside intervention. The results revealed that, compared to the control group, the concentration of fatty acyl-CoA increased, while the concentrations of acetyl-CoA and FFA significantly decreased, and ATP concentrations were reduced in GC cells treated with salidroside (**Figure 3C**). Once the concentration of fatty acyl-CoA don’t change, FFA and acetyl-CoA may also remain unchanged, which illustrates the importance of the substrate fatty acyl-CoA. Collectively, these data demonstrate that salidroside disrupts lipid metabolism and impairs energy homeostasis in GC cells through miR-1343-3p-mediated suppression of ACOT11.

### 3.4 Salidroside inhibits GC cell proliferation *in vitro* through modulating miR-1343-3p/ACOT11/FFA lipid metabolism signaling pathway

**Figure 4:**
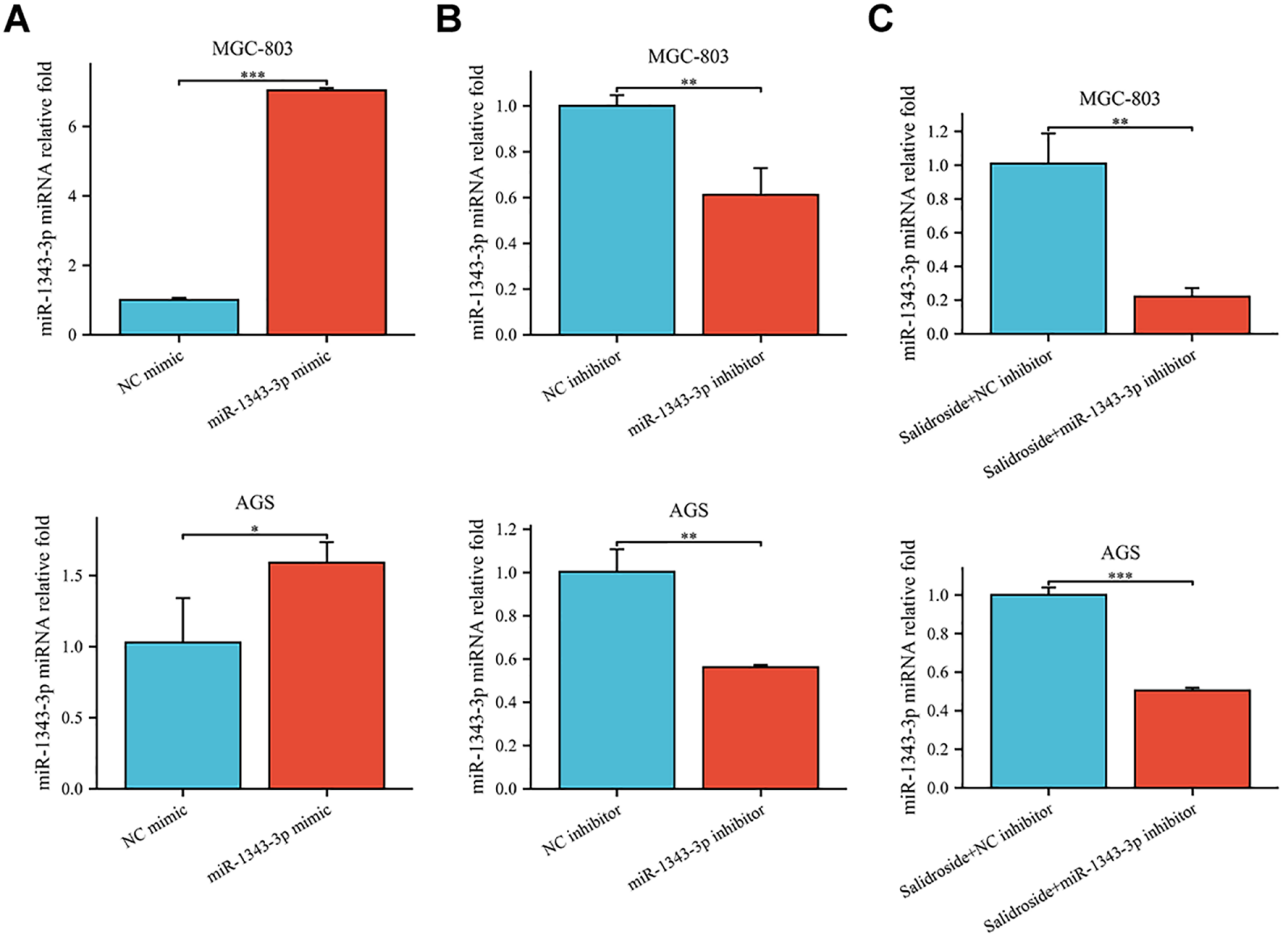
RT-qPCR was used to assess the transfection efficiency of miR-1343-3p mimic and miR-1343-3p inhibitor in human GC cells. **(A)**. Compared to the NC mimic group, the relative expression of the miR-1343-3p gene was measured in the miR-1343-3p mimic group (*n*=3); **(B)**. Compared to the NC inhibitor group, the relative expression of the miR-1343-3p gene was measured in the miR-1343-3p inhibitor group (*n*=3); **(C)**. Compared to the Salidroside + NC inhibitor group, the relative expression of the miR-1343-3p gene was measured in the Salidroside + miR-1343-3p inhibitor group (*n*=3); *p< 0.05; **P< 0.01, ***P< 0.001. MGC-803: Salidroside is 6 μmol/mL; AGS: Salidroside is 0.04 μmol/mL.

**Figure 5:**
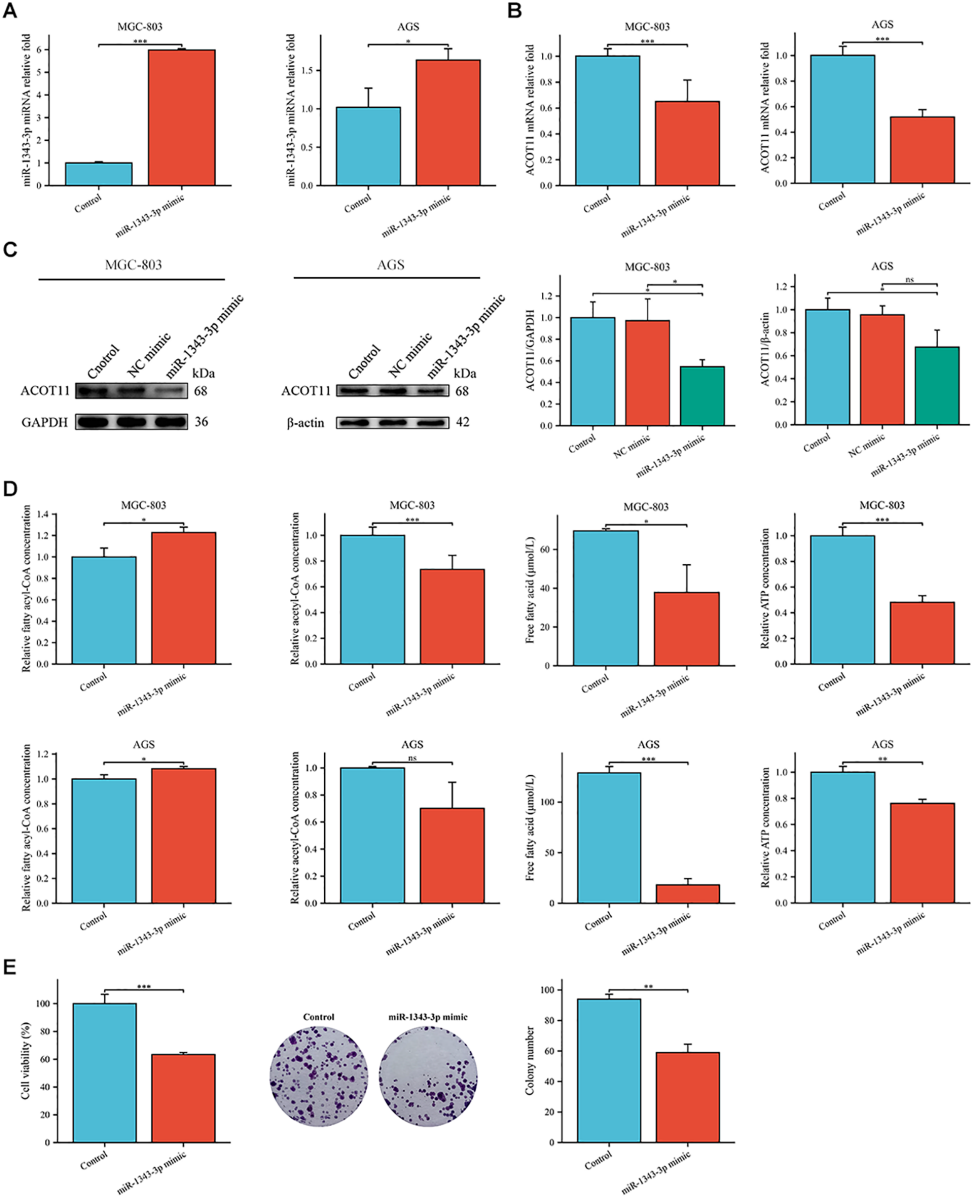
Gene expression profiles, protein abundance, and metabolite levels in GC cells following transfection with miR-1343-3p mimic. **(A, B)**. RT-qPCR analysis of the gene expression levels of miR-1343-3p and ACOT11 in GC cells (*n*=3); **(C)**. Western blot analysis of the protein expression levels of ACOT11 in GC cells (*n*=3); **(D)**. ELISA, microplate assay, and luciferase assay were used to measure the concentration changes of fatty acyl-CoA, acetyl-CoA, FFA, and ATP in GC cells (*n*=3); **(E)**. CCK-8 and colony formation assays were performed to assess the proliferation of MGC-803 cells (*n*=3); *P< 0.05, **P< 0.01, ***P< 0.001. ns, not significant.

**Figure 6:**
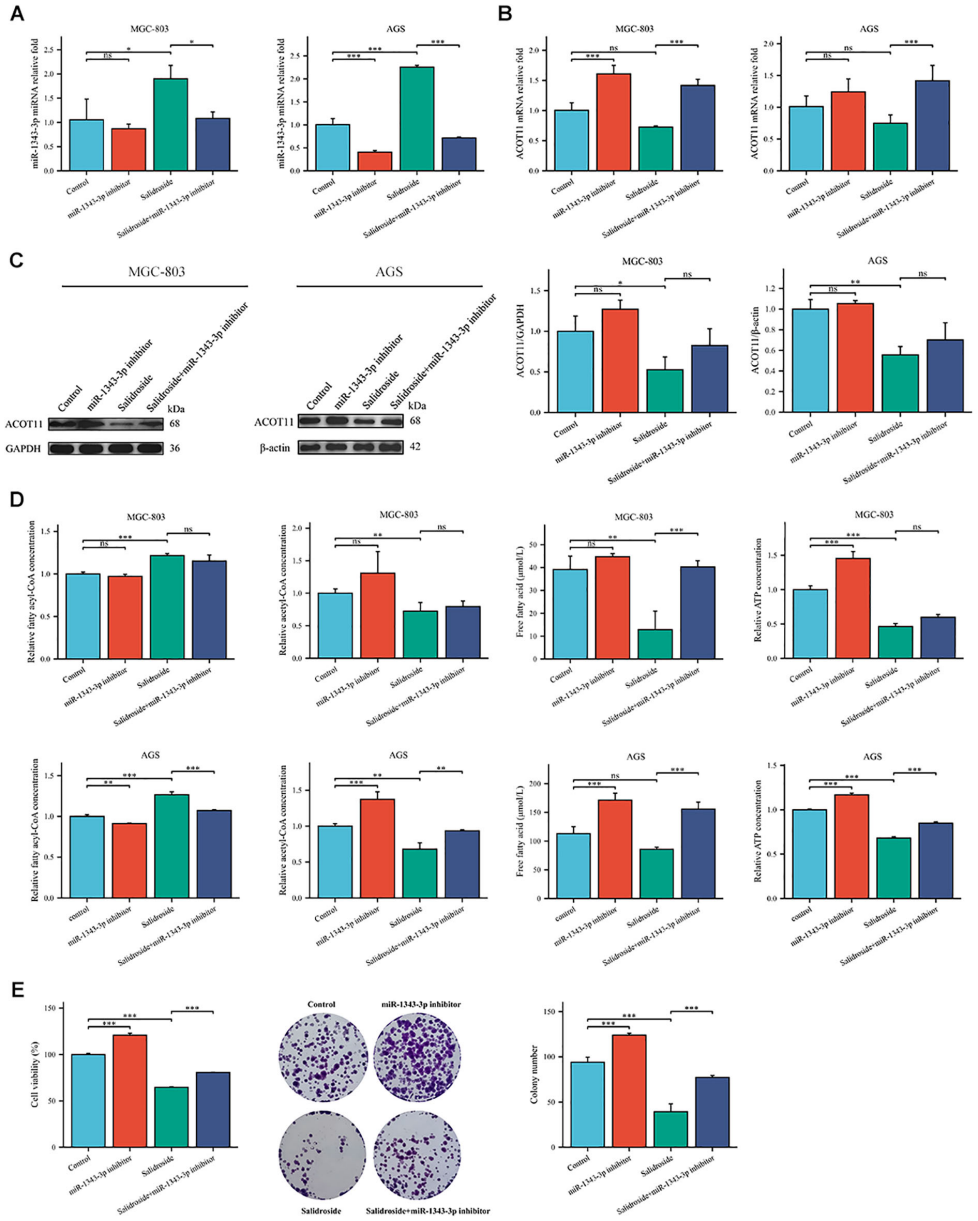
Gene expression profiles, protein abundance, and metabolite levels in GC cells following transfection with miR-1343-3p inhibitor. **(A, B)**. RT-qPCR analysis of the gene expression levels of miR-1343-3p and ACOT11 in GC cells (*n*=3); **(C)**. Western blot analysis of the protein expression levels of ACOT11 in GC cells (*n*=3); **(D)**. ELISA, microplate assay, and luciferase assay were used to measure the concentration changes of fatty acyl-CoA, acetyl-CoA, FFA, and ATP in GC cells (*n*=3); **(E)**. CCK-8 and colony formation assays were performed to assess the proliferation of MGC-803 cells (*n*=3); *P< 0.05, **P< 0.01, ***P< 0.001. ns, not significant. MGC-803: Salidroside is 6 μmol/mL; AGS: Salidroside is 0.04 μmol/mL.

To further investigate the effect target of ACOT11 on miR-1343-3p and determine the miR-1343-3p/ACOT11/FFA lipid metabolism signaling pathway as a potential effect target of salidroside’s anti-tumor effect, miR-1343-3p mimic and miR-1343-3p inhibitor were designed and transfected into GC cells. After 48 hours of transfection, CCK-8, colony formation, RT-qPCR, western blot, ELISA, microplate assay, and luciferase assays were performed to assess the effects of miR-1343-3p over-expression or inhibition on cell proliferation. Additionally, the ACOT11 expression level and associated metabolic impacts were examined via enzymatic measurement of fatty acyl-CoA, and downstream products acetyl-CoA and FFA in human GC cells. The results demonstrated that miR-1343-3p expression was significantly upregulated in GC cells transfected with the miR-1343-3p mimic compared to the NC mimic group, confirming efficient transfection (**Figure 4A**). Conversely, transfection with miR-1343-3p inhibitor in GC cells resulted in significant downregulation of miR-1343-3p expression in both the miR-1343-3p inhibitor group and Salidroside+miR-1343-3p inhibitor group (**Figures 4B**, 4C). Following transfection with the miR-1343-3p mimic, miR-1343-3p expression level was markedly elevated in GC cells (**Figure 5A**), whereas both mRNA and protein levels of ACOT11 were significantly suppressed (**Figures 5B, C**). The accumulation of fatty acyl-CoA resulted in diminished FFA production, accompanied by reduced intracellular FFA concentrations and impaired ATP generation (**Figure 5D**), culminating in the suppression of GC cell proliferation (**Figure 5E**). Conversely, Following transfection with the miR-1343-3p inhibitor, miR-1343-3p expression was significantly suppressed in GC cells (**Figure 6A**), while the gene and protein expression of ACOT11 was significantly upregulated (**Figures 6B, C**). The reduction of fatty acyl-CoA led to elevated FFA biosynthesis, resulting in increased intracellular FFA accumulation and subsequent augmentation of ATP production (**Figure 6D**), promoting GC cell proliferation (**Figure 6E**).

**Figure 7:**
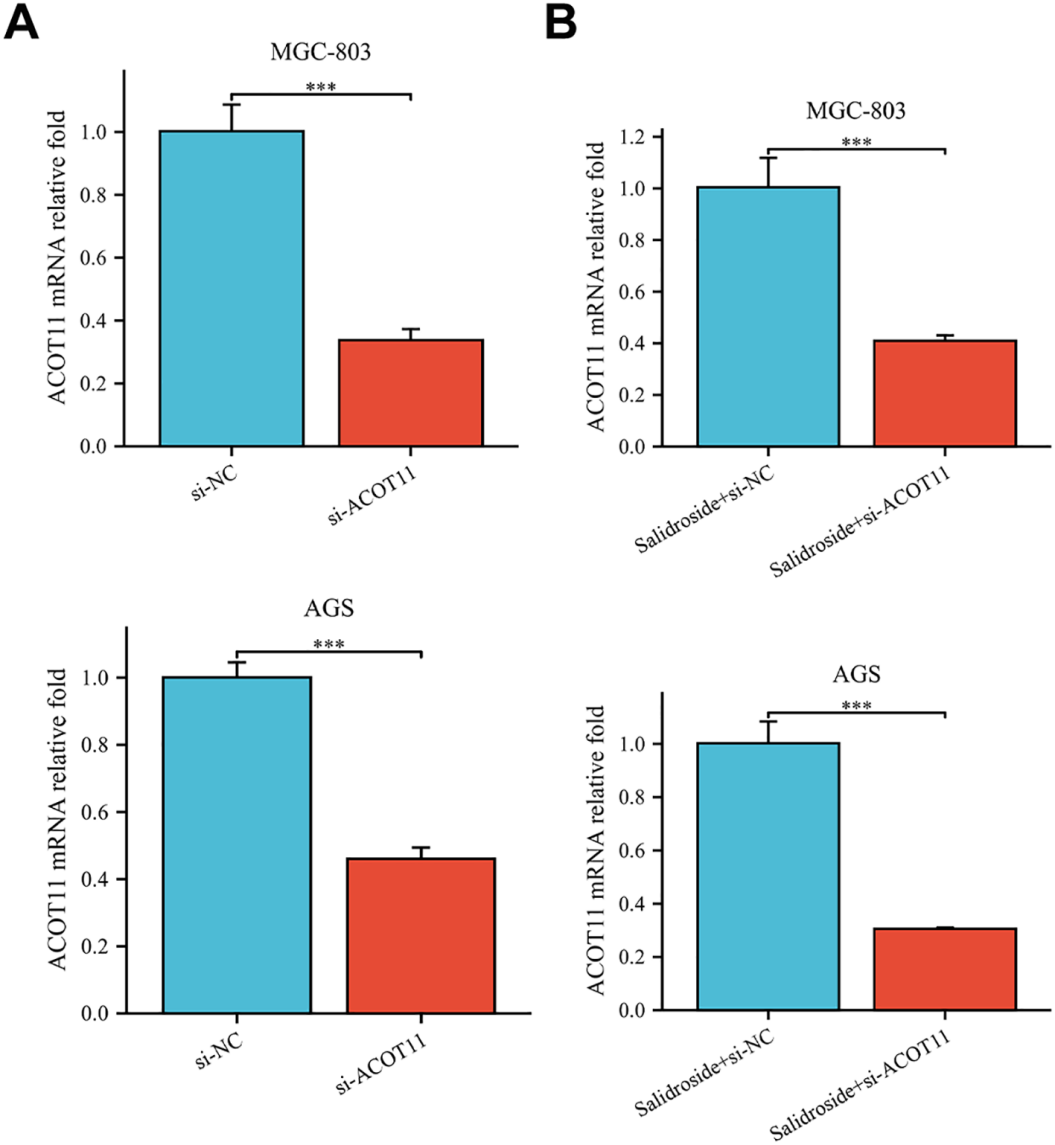
RT-qPCR analysis confirmed successful si-ACOT11 transfection in human GC cells. **(A)**. Compared to the si-NC group, the relative expression of the ACOT11 gene was measured in the si-ACOT11 group (*n*=3); **(B)**. Compared to the Salidroside + si-NC group, the relative expression of the ACOT11 gene was measured in the Salidroside + si-ACOT11 group (*n*=3); ***P< 0.001. MGC-803: Salidroside is 6 μmol/mL; AGS: Salidroside is 0.04 μmol/mL.

**Figure 8:**
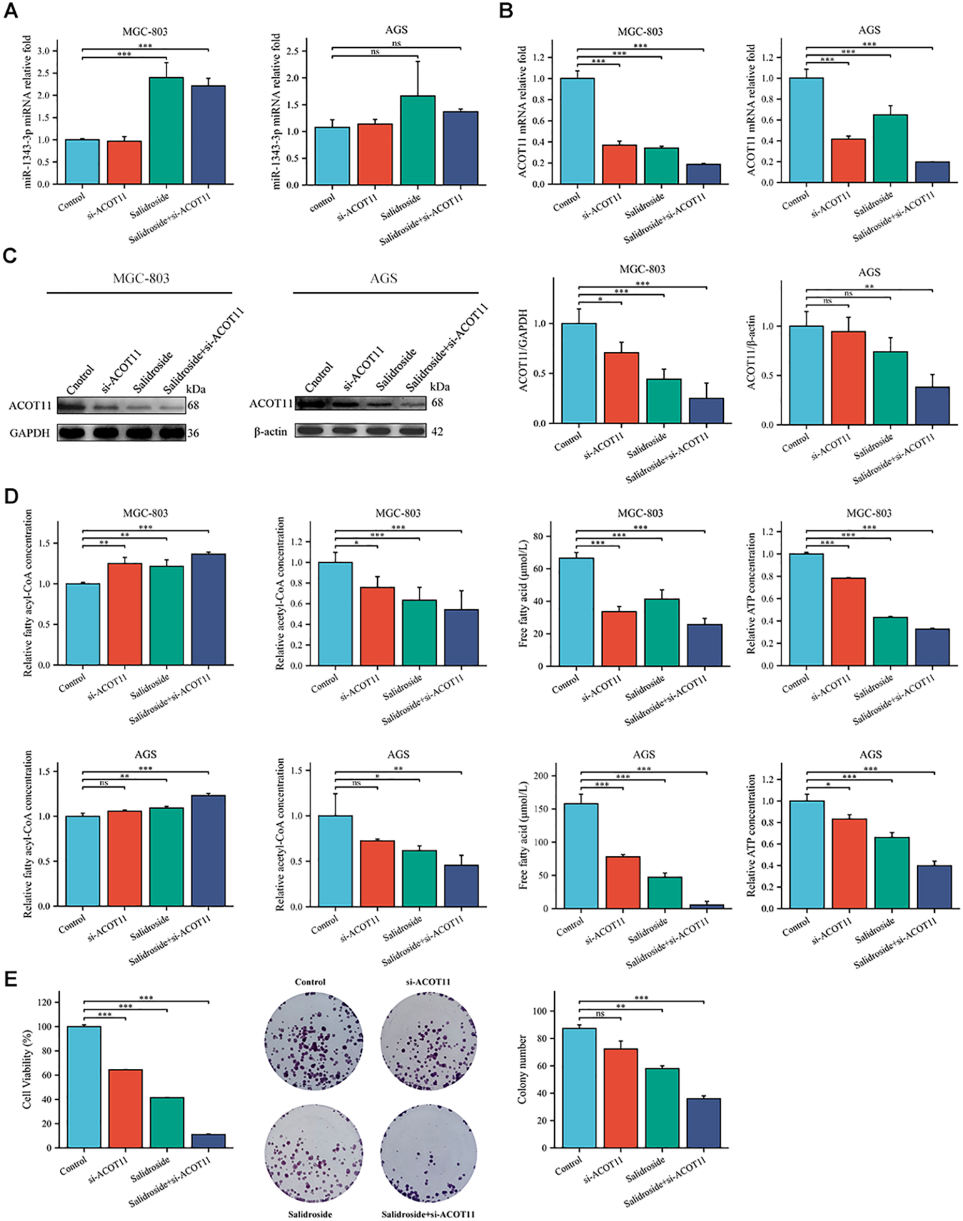
Gene expression profiles, protein abundance, and metabolite levels in GC cells following transfection with si-ACOT11. **(A, B)**. RT-qPCR analysis of the gene expression levels of miR-1343-3p and ACOT11 in GC cells (*n*=3); **(C)**. Western blot analysis of the protein expression levels of ACOT11 in GC cells (*n*=4); **(D)**. ELISA, microplate assay, and luciferase assay were used to measure the concentration changes of fatty acyl-CoA, acetyl-CoA, FFA, and ATP in GC cells (*n*=3); **(E)**. CCK-8 and colony formation assays were performed to assess the proliferation of MGC-803 cells (*n*=3); *P< 0.05, **P< 0.01, ***P< 0.001. ns, not significant. MGC-803: Salidroside is 6 μmol/mL; AGS: Salidroside is 0.04 μmol/mL.

siRNA targeting ACOT11 (si-ACOT11) was designed and transfected into GC cells using lipofection. After 48 hours of transfection, RT-qPCR was performed to assess ACOT11 expression levels. The results demonstrated successful transfection, with both the si-ACOT11 and Salidroside+si-ACOT11 groups showing significant downregulation of ACOT11 expression compared to the si-NC and Salidroside+si-NC control groups (**Figure 7**). When GC cells were transfected with si-ACOT11 to knock down ACOT11, the expression of miR-1343-3p remained unchanged (**Figure 8A**), while the gene and protein expression of ACOT11 was significantly downregulated (**Figures 8B, C**). The accumulation of fatty acyl-CoA led to diminished free fatty acid (FFA) biosynthesis, resulting in decreased intracellular FFA accumulation and subsequent impairment of ATP generation (**Figure 8D**), consequently, the metabolic disruptions inhibited GC cell proliferation (**Figure 8E**). As shown in **Figure 6**, the combination of salidroside and miR-1343-3p inhibitor reversed the inhibitory effect of salidroside on metabolism and anti-proliferative effects in GC cells. As shown in **Figure 8**, the combined treatment of salidroside and si-ACOT11 synergistically enhanced the observed effects in GC cells, resulting in more significant metabolic and anti-proliferative outcomes. Our comprehensive bidirectional *in vitro* experimental results demonstrate that salidroside mediates downregulating ACOT11 by upregulation of miR-1343-3p, leading to the accumulation of fatty acyl-CoA, reduced production of downstream products acetyl-CoA, FFA, and ATP, interference with lipid metabolism and energy production in GC cells, and ultimately inhibiting the growth of GC cells.

### 3.5 Salidroside inhibits GC cell proliferation *in vivo* through the miR-1343-3p/ACOT11/FFA lipid metabolism signaling pathway

**Figure 9:**
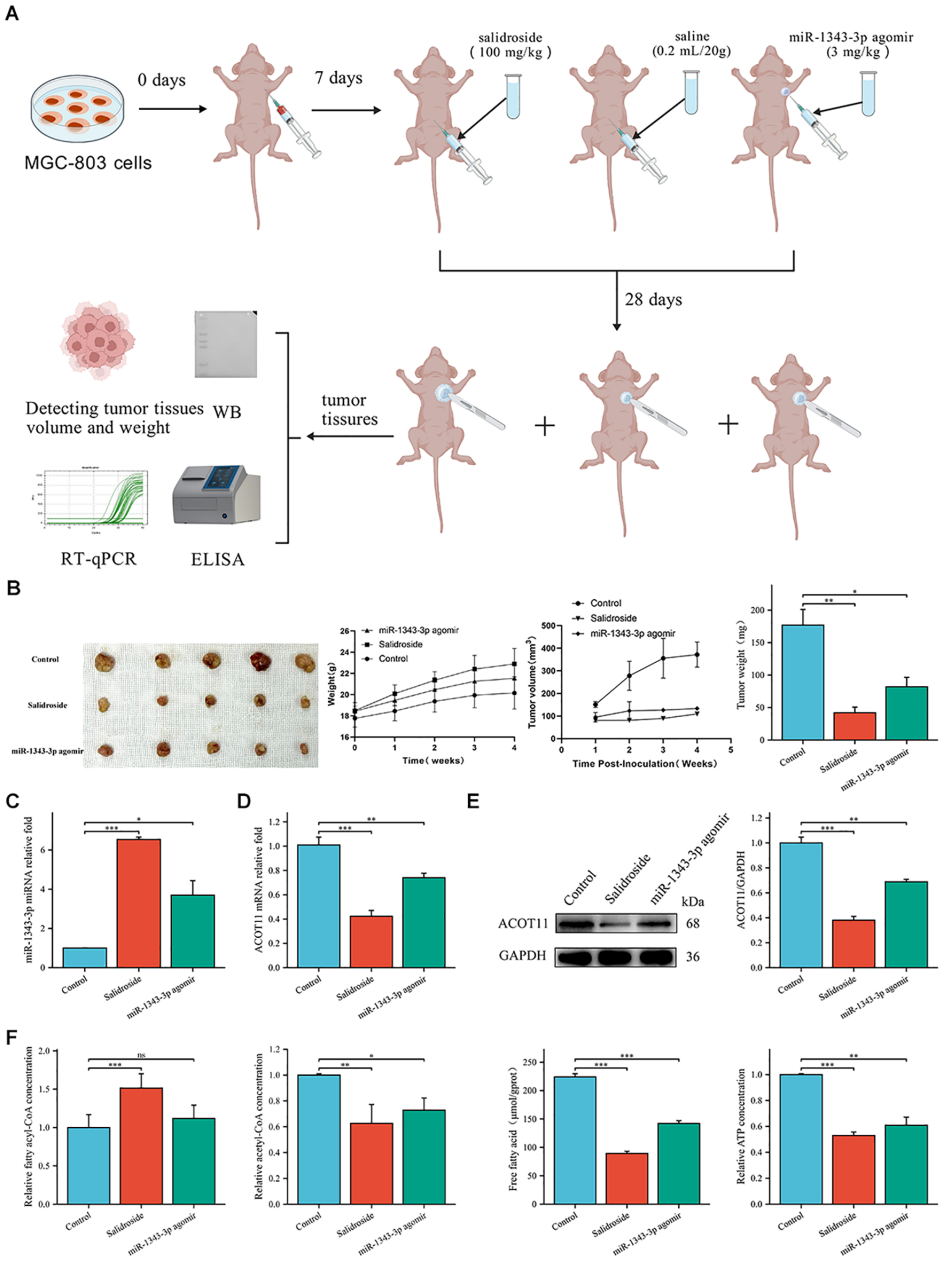
Salidroside inhibits GC progression through miR-1343-3p-mediated ACOT11 suppression and lipid metabolic disruption in xenograft tumors. **(A)**. *In vitro* experimental design route. Using MGC-803 cells to establish a gastric tumor-bearing nude mouse model, and intervention treatment was initiated 7 days after inoculation with MGC-803 cells. The experiment was divided into a control group (intraperitoneal injection of saline 3 mg/kg), a salidroside group (intraperitoneal injection of salidroside 100 mg/kg), and a miR-1343-3p overexpression group (local injection of miR-1343-3p agonir 3 mg/kg into tumor tissues). On the 28th day, tumor tissues were collected, and tumor volume and weight were measured. RT-qPCR, Western Blot, ELISA, and other experiments were conducted to detect changes in various genes, proteins, and metabolites. **(B)**. The volume, weight of tumor tissue and body weight of nude mice (*n*=5); **(C, D)**. RT-qPCR analysis of the gene expression levels of miR-1343-3p and ACOT11 in GC cells (*n*=3); **(E)**. Western blot analysis of the protein expression levels of ACOT11 in GC cells (*n*=3); **(F)**. ELISA, microplate assay, and luciferase assay were used to measure the concentration changes of fatty acyl-CoA, acetyl-CoA, FFA, and ATP in GC cells (*n*=3); *P< 0.05, **P< 0.01, ***P< 0.001. ns, not significant.

*In vivo* experiments using tumour-bearing nude mice confirmed that salidroside and over-expressed miR-1343-3p have a certain inhibitory effect on GC cells (**Figure 9A**). Tumor volume and weight were significantly reduced in all salidroside treatment groups relative to controls (**Figure 9B**), demonstrating superior anti-tumour effect. Compared to the control group, salidroside treatment resulted in increased expression levels of miR-1343-3p (**Figure 9C**), while ACOT11 was downregulated at both the transcription and protein levels (**Figures 9D, E**). The treatment induced significant fatty acyl-CoA accumulation while concurrently reducing acetyl-CoA and FFA levels, ultimately leading to impaired ATP generation (**Figure 9F**). Furthermore, after overexpressing miR-1343-3p, the gene and protein expression levels of ACOT11 and the production of downstream metabolites were consistent with the aforementioned results. The in vivo experimental results indicate that salidroside regulates ACOT11 through miR-1343-3p to inhibit lipid metabolism and proliferation in tumour tissues.

## 4 Discussion

GC is the most common malignant tumor of the digestive system, primarily associated with genetic factors (family history, especially first-degree relatives), poor lifestyle habits (obesity, smoking, sedentary behavior, high-sugar and high-fat diets), environmental factors (geographical distribution, soil, and water quality), chronic inflammatory stimulation, and Helicobacter pylori infection (20, 21). Clinical follow-up data demonstrate a striking disparity in 5-year survival outcomes: while early-stage GC patients exhibit favorable prognosis with >90% survival, advanced-stage patients show markedly poorer outcomes (<30% survival) even with surgical intervention (22). Due to the hidden and lack of specific signs in early-stage GC, invasive and radiation-based diagnostic methods such as endoscopic biopsy and imaging, along with the low sensitivity of traditional tumor markers like carcinoembryonic antigen (CEA) and carbohydrate antigen 199 (CA199), result in low detection rates (23–25). Consequently, most patients (>70%) are diagnosed beyond the early stage, often at an advanced stage, missing the optimal timing and methods for treatment, and ultimately relying on chemotherapy and radiotherapy (26). Despite the availability of multiple chemotherapeutic regimens for GC, their clinical utility remains limited by transient therapeutic responses and high rates of tumor recurrence/metastasis, which collectively contribute to poor long-term survival outcomes. Salidroside, the primary active component of Rhodiola, has been shown to inhibit GC growth (5). Salidroside is a multi-effect, multi-target biological regulator that influences multiple signaling targets and biological effects, thereby modulating the proliferation, invasion, and metastasis of various cancers (4). However, research on salidroside’s regulation of non-coding RNAs in tumor progression remains limited. Our study demonstrates through both *in vivo* and *in vitro* experiments that salidroside treatment significantly upregulates miR-1343-3p in GC cells. This up-regulation leads to targeted suppression of ACOT11 gene and protein expression, resulting in decreased hydrolysis of the fatty acyl-CoA substrate (with consequent accumulation), reduced generation of FFA and acetyl-CoA, and markedly diminished ATP production. These metabolic changes ultimately inhibit lipid metabolism and proliferation in GC cells.

Recent studies have found that abnormal lipid metabolism reprogramming provides nutrients and energy for tumors, primarily by increasing fatty acid uptake and synthesis to promote rapid cancer cell growth (27, 28). For instance, ATP citrate lyase and fatty acid synthase are often found overexpressed in gastric cancer tissues, where increased expression of these genes facilitates fatty acid synthesis, providing essential energy and structural support for tumor cell growth (29). Furthermore, studies have observed enhanced activity of key regulatory factors in fatty acid metabolism, such as sterol regulatory element-binding protein 1, in gastric cancer cells, which further promotes lipid synthesis and cellular proliferation1 (30). These alterations in gene expression not only reshape the metabolic state of tumor cells but may also influence immune cell function by modifying lipid components within the tumor microenvironment, thereby fostering cancer progression (31). Dysregulated lipid metabolism can also alter cell membrane composition, gene expression, signaling pathway activity, and downstream cellular functions, directly influencing cancer development and progression (32). Advances in molecular biology and metabolomics have revealed that cancer initiation, progression, and metastasis are closely linked to non-coding RNAs and lipid metabolism. Competing endogenous RNA (ceRNA) is a hypothesized mechanism of mutual regulation between RNA molecules through shared miRNA response elements. miRNAs, as core components of ceRNA regulation, can act as tumor suppressors or oncogenes, modulating lipid metabolism in tumor tissues. Their aberrant expression plays a critical role in GC development, influencing processes such as proliferation, migration, and apoptosis (33, 34). For example, Hcp5 sponges miR-3619-5p and upregulates PPARGC1α, increasing the transcriptional complex PGC1α/CEBPB to induce CPT1 transcription, thereby promoting fatty acid oxidation and GC progression (35). Mirtronic miR-4646-5p can facilitate GC metastasis by regulating ABHD16A and the metabolite lysophosphatidylserine (36). As a tumor suppressor, miR-1343-3p can inhibit the expression and activation of the GC oncogene TEAD4 to some extent (19), and can also promote autophagy in thyroid cancer cells through ATG7 (37). Related studies showed that miR-1343-3p can be suppressed by LINC01559 adsorption, leading to PGK1 up-regulation and activation of the PI3K/AKT pathway, thereby promoting GC proliferation and migration (38).

Our previous research confirmed that salidroside significantly upregulates the tumor suppressor miR-1343-3p in GC cells, which is nearly non-expressed in the control group but markedly induced after salidroside treatment (18). Through integrated bioinformatics analysis, including miRNA-mRNA target prediction and functional enrichment studies (GO/KEGG) (18), we identified ACOT11 as a key regulator of FFA metabolism that is both significantly downregulated and directly modulated by miR-1343-3p. ACOT11 expression is negatively correlated with miR-1343-3p. Acyl-CoA thioesterases (ACOTs) are an essential enzyme family in mammalian fatty acid metabolism, hydrolyzing fatty acyl-CoA in mitochondria to form FFA and acetyl-CoA. FFAs undergo β-oxidation to produce ATP, regulating critical cellular metabolic processes such as lipid biosynthesis and signal transduction (39). Current research on ACOT11 in tumor metabolism remains limited, with only a few other ACOT family members reported to participate in cancer metabolic reprogramming. For instance: ACOT8 overexpression correlates with lung adenocarcinoma metastasis and may mediate intracellular lipid metabolism (40). In hepatocellular carcinoma, ACOT12 regulates cellular acetyl-CoA levels and histone acetylation to promote epithelial-mesenchymal transition and metastasis (41). ACOT13 and ACOT15 have also been implicated in modulating hepatic lipid metabolism (42, 43). ACOT11 is highly expressed in lung adenocarcinoma cells, promoting proliferation, migration, and invasion (44), suggesting its role in cancer progression. However, the mechanism of ACOT11 in GC remains unclear.

This study, employing Pearson correlation and genetic locus analysis, revealed a significant association between ACOT11 and miR-1343-3p in GC cells. Further RIP assays confirmed the interaction between miR-1343-3p and ACOT11, indicating that salidroside likely inhibits GC cell proliferation by down-regulating ACOT11 through miR-1343-3p targeting, thereby interfering with lipid metabolism and energy production. Subsequent mechanistic investigations demonstrated that salidroside treatment significantly upregulated miR-1343-3p expression while concurrently downregulating ACOT11 at both transcriptional and translational levels. *In vivo* studies confirmed that salidroside and miR-1343-3p agomir reduced tumor size, volume, and weight, upregulated miR-1343-3p expression, downregulated ACOT11 at the gene and protein levels, increased fatty acyl-CoA concentration, and decreased acetyl-CoA, FFA, and ATP concentrations. These results indicate that salidroside affects GC proliferation by regulating the miR-1343-3p/ACOT11/FFA lipid metabolism signaling pathway. Yang et al. developed a novel nano-platform encapsulating LINC00958 siRNA for the systemic treatment of liver cancer. Histopathological examination of mouse liver HE staining and blood biochemical tests confirmed that si-LINC00958 had no obvious toxic or side effects, which holds significant implications for further clinical trials (45). This suggests that regulatory factors such as miR-1343-3p and ACOT11, which are associated with GC, may also become key targets for diagnosis and treatment.

Although this study confirms that salidroside exhibits strong anti-cancer effects in both cancer cells and animal tumor models, it lacks sufficient clinical trial evidence to demonstrate its clinical efficacy, safety, stability, and the persistence of therapeutic outcomes; additionally, extensive experiments are needed to validate the degree of drug resistance induced during treatment. Furthermore, the complexity of salidroside’s mechanism via the miRNA-mRNA signaling axis may introduce hidden factors influencing therapeutic efficacy. Meanwhile, the current research has only established this mechanism in a single GC cell line, demonstrating salidroside’s crucial role in regulating lipid metabolism and suppressing tumor growth through up-regulation of the important tumor-suppressing factor miR-1343-3p. This limitation necessitates further investigation. Our research group will subsequently validate these findings across multiple GC cell lines to confirm the universality of these results and advance this line of research.

Existing studies indicate that salidroside may upregulate miR-195, modulating the Protein kinase B (AKT) and MEK/ERK signaling pathways, or enhance miR-103-3p to target the Marginal zone B-and B1-cell-specific protein (Mzb1), thereby suppressing lung cancer proliferation and metastasis (46, 47). Additionally, research has reported that salidroside may inhibit nasopharyngeal carcinoma proliferation by up-regulating miR-4262, which targets Glucose-regulated protein 78 (GRP78) (48) The current study has systematically elucidated for the first time the mechanism by which salidroside inhibits gastric cancer through lipid metabolism pathways, providing a theoretical basis for salidroside to inhibit gastric cancer through miRNA. Looking forward, we propose to expand this research by examining salidroside’s regulation of non-coding RNAs and establishing a molecular network of salidroside anti-tumor activity and miRNA regulation in gastric cancer. Future studies should explore salidroside’s multi-target, multi-effect mechanisms against GC through various pathways including glucose metabolism, autophagy, methylation and cell death processes. A deeper investigation into the interaction network between miR-1343-3p and its target mRNA genes under salidroside regulation will provide novel theoretical insights into GC pathogenesis. In a word, this work provides a new research direction for the diagnosis and treatment of GC, making it possible to develop novel molecular markers for the treatment of GC.

## 5 Conclusions

This study establishes a novel anti-tumor mechanism of salidroside in GC through the miR-1343-3p/ACOT11/FFA metabolic axis. We demonstrate that salidroside potently induces miR-1343-3p expression, which directly targets and suppresses ACOT11, resulting in significant downregulation at both mRNA and protein levels. This molecular regulation leads to profound metabolic disruption characterized by fatty acyl-CoA accumulation, impaired hydrolysis, and subsequent depletion of downstream metabolites and ATP production. This study holds significant importance for understanding lipid metabolism reprogramming in GC cells and cancer progression, offering substantial potential for the development of targeted therapies.

## Data Availability

The raw data supporting the conclusions of this article will be made available by the authors, without undue reservation.
